# Effect of Temperature Conditions on the Physicochemical Quality of Aged Black Garlic

**DOI:** 10.3390/foods13233974

**Published:** 2024-12-09

**Authors:** Jung-Hye Shin, Min-Jung Kang, Bo Hyun Lee, Dawon Kang

**Affiliations:** 1 Namhae Garlic Research Institute, Namhae-gun 52430, Republic of Korea; whanbee@hanmail.net (J.-H.S.); jung-75@hanmail.net (M.-J.K.); 2 Department of Physiology, Institute of Medical Sciences, College of Medicine, Gyeongsang National University, Jinju 52727, Republic of Korea; blee@gnu.ac.kr; 3 Department of Convergence Medical Science, Gyeongsang National University, Jinju 52727, Republic of Korea

**Keywords:** alliin, black garlic, γ-glutamyl-S-allylcysteine, S-allylcysteine, temperature

## Abstract

This study investigates the effects of different temperature conditions on the quality of black garlic (BG) during the aging process. Two temperature protocols were employed: gradual heating and cooling (GHC), where the temperature was slowly raised from 45 °C to 77 °C and then lowered to 59 °C at a rate of 1 °C per hour, and rapid heating and cooling (RHC), where the temperature was quickly raised from 45 °C to 85 °C and then lowered to 56 °C at a rate of 1 °C every 30 min. Changes in surface color, hardness, moisture, pH, fructose, total polyphenol content (TPC), and key sulfur compounds such as alliin, S-allylcysteine (SAC), and γ-glutamyl-S-allylcysteine (γ-GSAC) were analyzed. Our findings showed that GHC led to a higher increase in TPC and fructose content by the 15th day compared to RHC. In contrast, RHC retained significantly higher SAC concentrations, approximately 1.7 times that of GHC, by the end of the aging period. Surface color changes, particularly in lightness and redness, were more pronounced under GHC, while RHC demonstrated superior moisture retention. These findings indicate that GHC is better suited for products prioritizing polyphenols and sugars, while RHC is more optimal for SAC-enriched BG. This study provides valuable insights into optimizing BG production for diverse food and medicinal applications through precise temperature modulation.

## 1. Introduction

Garlic (*Allium sativum* L.) is a widely cherished seasoning and traditional medicinal herb renowned globally for its health benefits, which are well-supported by scientific research [1]. The medicinal properties of garlic are primarily due to its organosulfur compounds (OSCs), such as alkenyl-cysteine sulfoxides, polysulfides, ajoenes, and vinyldithiins. These compounds exhibit various health-promoting effects, including anticancer, antimicrobial, antifungal, antioxidant, antidiabetic, blood-pressure-lowering, and immune-enhancing activities [2,3]. Despite these benefits, the strong odor and taste of garlic—largely attributed to its OSCs—often discourage consumption. As a result, various processing methods have been explored to reduce its pungency and improve its palatability [4]. Initially, traditional cooking methods like steaming, roasting, and frying were used, but these approaches limited the potential of garlic as a functional health food.

In response, the food industry has explored alternative methods such as prolonged aging in ethanol or processing with hot water extraction to both mellow the strong flavor and enhance the extraction of active ingredients [5]. Before the advent of black garlic (BG) roughly two decades ago, these methods dominated garlic processing. The journey of BG as a commercial product began in 2004 with the filing of the first patent in Japan [JP-0347949, 1 December 2004], followed by a subsequent patent in Korea in 2007 [KR-1020070013660, 9 February 2007]. Since then, BG has gained popularity in several Asian countries, including South Korea, Japan, China, and Thailand, becoming an important subject of research across various fields [6].

BG is produced by aging raw garlic under controlled conditions of high temperature (50–90 °C) and humidity (70–90%) for 10 to 60 days without the use of any additives [7,8,9,10]. Previous studies have aged BG at 70 °C and 80% humidity for 30 days, 50 °C and 70% humidity for 28 days, or 80 °C and 80% humidity for 14 days, each using a fixed single temperature throughout the process [9,10]. During this process, the Maillard reaction transforms the garlic’s color to a distinctive dark brown or black. Over the past 20 years, studies have highlighted BG’s enhanced physiological activities, such as antioxidant, anticancer, hepatoprotective, immune-boosting, anti-inflammatory, and anti-allergic effects, as well as its ability to regulate glucose and lipid metabolism—effects that surpass those of fresh garlic [11,12,13,14]. The transformation of raw garlic into BG is significantly influenced by the applied heat treatment, which alters its chemical composition. Even when starting with identical raw materials, factors such as the method of temperature adjustment, the range of temperatures, and the duration of heating play critical roles in determining the final composition of BG [8,15]. A deeper understanding of these variables is essential for discerning the quality differences in BG. However, the effect of subtle adjustments in aging parameters on BG’s primary components remains underexplored.

In this study, we investigated the physicochemical properties and bioactive components of BG under different temperature conditions during a 15-day aging period, following a patented procedure [KR Patent 10-1989494]. The 15-day duration was selected as it represents the period before S-allylcysteine (SAC) concentration peaks and begins to decline significantly [8,16]. The maximum temperature was set at 75 °C or higher, as temperatures above 70 °C accelerate the aging process compared to 60 °C, leading to tissue softening and increased levels of sugars and browning substances. The garlic begins to soften around day 4 of the aging process. To preserve the distinctive texture of BG, the temperature is gradually reduced starting on day 4. Conversely, at 60 °C, SAC content increases but requires a longer aging period [16]. For commercial BG production, adopting an aging method that balances aging duration, sugar content, texture, and the preservation of functional components would be highly advantageous.

This study differentiates itself from previous studies by employing detailed temperature adjustments during the aging process rather than a single fixed temperature. We produced BG using two distinct approaches: one involving a gradual temperature reduction from a peak of 77 °C (gradual heating and cooling, GHC), and the other featuring a more abrupt shift from 85 °C (rapid heating and cooling, RHC), followed by temperature stabilization for specific durations. The results of these two methods were then systematically compared to assess the differences in BG characteristics.

## 2. Materials and Methods

### 2.1. Garlic Preparation and Black Garlic (BG) Production

Namdo garlic cultivar was used, which was grown in Namhae (Gyeongnam, Republic of Korea) from October to June, and each bulb weighed between 35 and 65 g. After harvesting, the garlic was dried in an air drying system at 40 °C for 10 days after removing the stems and leaves. Then, 60 kg of dried garlic bulbs were separated into 5 kg units and placed in covered steel trays (300 × 500 × 150 mm). These trays were then placed in two constant temperature and humidity chambers (JSRH-500CPL, JS Research, Gongju, Republic of Korea) for the aging process.

**Figure 1:**
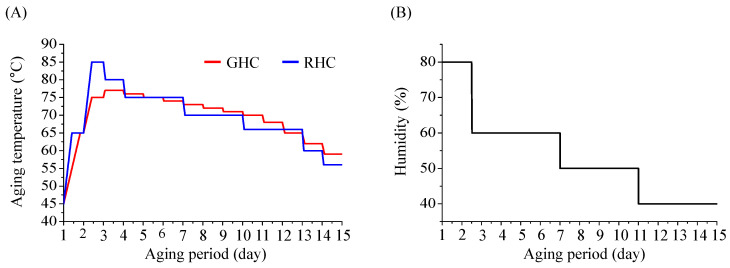
Aging conditions for black garlic (BG) manufacturing under different temperature protocols. (**A**) Temperature changes during the aging process. (**B**) Humidity changes during the aging process. The same humidity conditions were applied to both GHC and RHC groups (80%-60%-50%-40%). GHC and RHC refer to gradual heating and cooling and rapid heating and cooling, respectively.

Two distinct aging temperature protocols were employed (Figure 1). Gradual heating and cooling (GHC) involved a controlled temperature adjustment at a rate of 1 °C per hour, starting at 45 °C, gradually rising to a peak of 77 °C, and then decreasing to 59 °C. Rapid heating and cooling (RHC) employed faster temperature modulation, starting at 45 °C, increasing to 85 °C, and then decreasing to 56 °C, with adjustments of 1 °C every 30 min. Samples were collected on days 1, 2, 3, 4, 5, 7, 8, 10, 11, 13, and 15, corresponding to the temperature change intervals. Approximately 180–250 g of garlic was sampled from the center of each tray, peeled, and prepared for analysis. The aging process was replicated thrice under identical conditions.

### 2.2. Surface Color and Hardness Analysis

For each test, over 20 peeled garlic cloves were prepared. Garlic samples were sliced to a thickness of 15 mm after peeling, and their surface color was measured using a colorimeter (Ultra Scan VIS, Hunter Associates Laboratory Inc., Reston, VA, USA). The device was calibrated with a standard white reflector plate (L* = 99.4, a* = 0.12, b* = 0.04) before measurement. Lightness (L*), redness (a*), and yellowness (b*) values were determined using the Hunter color system (*n* = 10). The color difference (ΔE*) compared to raw garlic (control) was calculated using the following formula.

$$\Delta E^{*}=\sqrt{{\left(L_{sample}^{*}-L_{control}^{*}\right)}^{2}+{\left(a_{sample}^{*}-a_{control}^{*}\right)}^{2}+{\left(b_{sample}^{*}-b_{control}^{*}\right)}^{2}}$$

The hardness test was conducted to measure the texture changes in BG during the aging process. The principle of the hardness test involved applying a consistent force to the surface of the BG using a texture analyzer equipped with a compression probe (TAXT express, Stable Microsystems Ltd., Godalming, UK). The maximum force required to compress the garlic to a predetermined distance was recorded as the hardness value. This method quantifies the firmness or softness of the black garlic. The analysis conditions were as follows: a 4 mm diameter probe was used, with a pre-test speed of 1.0 mm/s, a trigger force of 50.0 g, a test speed of 5.0 mm/s, a test distance of 5.0 mm, and a single test cycle.

### 2.3. Moisture and pH Measurements

Moisture content was determined using a moisture analyzer (MB25, OHAUS, Zurich, Switzerland) with 1 g of crushed garlic paste, following the protocol described previously [8]. For pH measurement, 5 g of crushed garlic were diluted to 50 mL with distilled water, vortexed, filtered, and analyzed using a pH meter (Model 720, Thermo Orion, Waltham, MA, USA).

### 2.4. Fructose Content Analysis

The analysis methodology was the same as in the previous study [8]. Two grams of crushed garlic were mixed with 20 mL of distilled water and extracted using an ultrasonic extractor for 30 min. The resulting extract was centrifuged at 1200× *g* for 10 min (Combi-514R, Hanil, Seoul, Republic of Korea) and then filtered through a 0.45 μm syringe filter. Fructose levels were quantified using high-performance liquid chromatography (HPLC, Agilent 1260, Agilent, CA, USA) equipped with a Cosmosil Sugar-D analytical column (4.6 × 250 mm, Nacalai Tesque Inc., Kyoto, Japan), following the modified method of Ma et al. [17]. The column temperature was maintained at 30 °C, and the mobile phase consisted of a 30:70 mixture of water and acetonitrile, with a flow rate of 1 mL/min. A 10 μL aliquot of the sample was injected for analysis. Detection was carried out using an evaporative light scattering detector (ELSD, Agilent LT-ELSD G4128A, Agilent Technologies, Santa Clara, CA, USA), and fructose content was quantified using a standard calibration curve.

### 2.5. Total Phenolic Content (TPC) Determination

TPC was measured using the Folin-Ciocalteu method [18]. The process involved mixing filtrate with reagents, incubation, and calibration against a standard curve of gallic acid equivalent (GAE).

### 2.6. Alliin Content Measurement

Alliin content measurement was performed following the protocol described previously [8,19]. Five grams of ground garlic were combined with 30 mL of methanol adjusted to pH 3.0 using phosphoric acid and subjected to ultrasonic extraction for 10 min. The resulting extract was then filtered through a 0.22 μm syringe filter and analyzed using HPLC. An analytical YMC-Triart C18 column (3 × 150 mm, 5 μm; Thermo Scientific, Waltham, MA, USA) was utilized for the analysis. The mobile phase comprised two components: eluent A, consisting of 20 mM sodium phosphate monobasic dihydrate and 10 mM sodium 1-heptane-sulfonic acid, adjusted to pH 2.0 using 85% orthophosphoric acid, and eluent B, which was acetonitrile. Gradient elution was performed, starting with a ratio of 100:0 (A:B, *v*/*v*) and transitioning to 0:100 (A:B, *v*/*v*). The column temperature was kept constant at 30 °C, with a mobile phase flow rate of 0.4 mL/min. The sample injection volume was set at 10 μL, and UV detection was conducted at a wavelength of 208 nm. The concentration of alliin was quantified using a standard calibration curve prepared with alliin reference material (ChromaDex Inc., Irvine, CA, USA).

### 2.7. Analysis of S-Allylcysteine (SAC) and γ-Glutamyl-S-Allylcysteine (γ-GSAC)

Five grams of garlic were homogenized in 45 mL of distilled water, followed by ultrasonic extraction for 30 min. The mixture was initially filtered using filter paper (No. 2, ADVANTEC, Tokyo, Japan) and subsequently re-filtered through a 0.22 μm syringe filter. The resulting filtrate was analyzed for SAC and γ-GSAC simultaneously using HPLC-PDA-MS/MS (TSQ Quantum LC-MS/MS, Thermo Scientific, Waltham, MA, USA) [8]. An Agilent Zorbax SB-C18 analytical column (4.6 × 250 mm, 5 μm, Agilent Technologies) was utilized with a flow rate of 0.7 mL/min for the separation of target compounds. The mobile phase consisted of two components: A (0.1% formic acid in water) and B (acetonitrile). A linear gradient program was employed with the following profile: 0–7 min, 20% B; 7–14 min, 100% B; 14–17 min, 100% B; and 17–25 min, 1% B. Quantification of S-allylcysteine (SAC) and γ-glutamyl-S-allylcysteine (GSAC) was performed using Xcalibur 2.1 software (Thermo Scientific). Standards of SAC (Sigma-Aldrich Co., Saint Louis, MO, USA) and GSAC (US Pharmacopeia, Rockville, MD, USA) were analyzed under identical conditions to the samples. Retention times of the standards were used to identify SAC and γ-GSAC in the samples, and their concentrations were calculated based on respective calibration curves.

### 2.8. Statistical Analysis

Data are presented as means ± standard deviation (SD) from three independent experiments. Statistical significance was determined using one-way ANOVA and Duncan’s multiple range test using SPSS (v 18.0; IBM Co., Endicott, NY, USA), with a significance level set at *p* < 0.05. Differences between two groups were evaluated using a paired Student’s *t*-test (OriginPro 2020 software; OriginLab Corp., Northampton, MA, USA). Partial Least Squares Discriminant Analysis (PLS-DA) was applied to visualize differences among the samples. The quality of the PLS-DA models was assessed based on their goodness of fit (R_2_X, R_2_Y) and predictive ability (Q_2_Y), with cross-validation performed using a permutation test. To identify components that contributed significantly to the aging period, those with a Variable Importance in Projection (VIP) value greater than 0.5 were considered. The analytical data sets were analyzed using multivariate statistical analysis with SIMCA-P+ version 12.0.1 (Umetrics, Umeå, Sweden).

## 3. Results

### 3.1. Colorimetric Analysis of Garlic’s Surface Color Under Different Aging Temperatures

**Figure 2:**
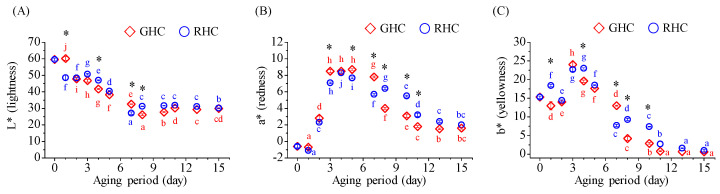
Changes in color metrics (lightness, redness, and yellowness) of BG during the 15-day aging process under two temperature protocols: GHC (gradual heating and cooling) and RHC (rapid heating and cooling). (**A**) Lightness (L*) values. (**B**) Redness (a*) values. (**C**) Yellowness (b*) values. Data are presented as mean ± SD from three independent experiments. Different letters indicate statistically significant differences within the same treatment group, as assessed by Duncan’s multiple range test (*p* < 0.05). A star symbol (*) indicates a significant difference between the two groups (GHC and RHC).

The surface color of raw garlic was analyzed using a colorimeter, which provided measurements for lightness (L), redness (a), and yellowness (b). For raw garlic, the initial values were L = 59.6 ± 0.8, a = −0.6 ± 0.2, and b = 15.3 ± 0.3. A significant reduction in the L value was observed on the 2nd day for GHC and on the 1st day for RHC, with a notable difference between the two groups (*p* < 0.05). This value continued to decline throughout the aging period, reaching 29.9 for GHC and 30.2 for RHC by the 15th day (Figure 2A). For redness (a), a sharp increase was observed on the 3rd day of aging, with values reaching 8.5 for GHC and 7.1 for RHC. The maximum redness was reached on the 5th day for GHC and the 4th day for RHC, followed by a steady decline over the remaining aging period (Figure 2B). The trend for yellowness (b) mirrored that of redness, with the lowest values recorded on the 15th day of aging (Figure 2C).

### 3.2. Changes in Hardness and Moisture of Garlic Subjected to Different Temperature Conditions During Aging

**Figure 3:**
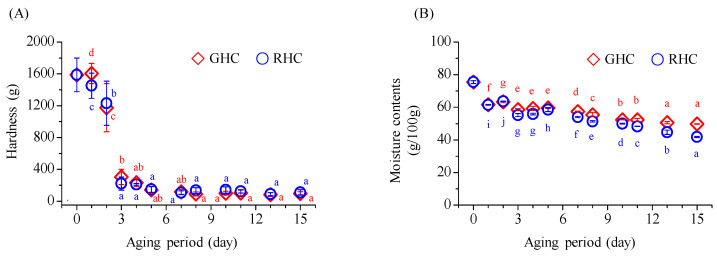
Changes in hardness and moisture content of BG during the 15-day aging period under two temperature protocols: GHC (gradual heating and cooling) and RHC (rapid heating and cooling). (**A**) Hardness values. (**B**) Moisture content values. Data are presented as mean ± SD from three independent experiments. Different letters indicate statistically significant differences within the same treatment group, as assessed by Duncan’s multiple range test (*p* < 0.05).

During the aging process of BG, a significant decrease in hardness was observed, reaching its lowest point by the 3rd day. Following this, the hardness continued to decrease, though the changes were not statistically significant (Figure 3A). The initial hardness of raw garlic was 1587.7 g, which softened considerably to 97.4 g in GHC and 110.8 g in RHC by the 15th day of aging. Regarding moisture content, a reduction to below 60.0 g/100.0 g occurred by the 3rd day, with a continuous decline throughout the aging period (Figure 3B). The final moisture content reached 50.6 g/100.0 g in BG from GHC and 41.8 g/100.0 g from RHC, representing a moisture loss of approximately 33–45% in the final BG product compared to the initial moisture content of raw garlic, which was 75.4%.

### 3.3. Changes in pH, Fructose, and Polyphenol Contents of Garlic Subjected to Different Temperature Conditions During Aging

**Figure 4:**
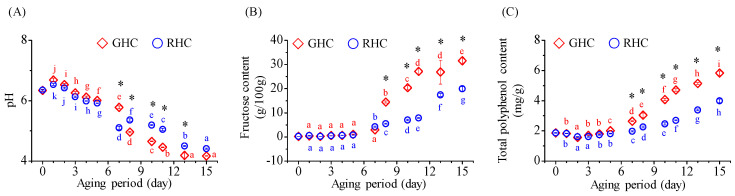
Changes in pH, fructose content, and total polyphenol content of BG during the 15-day aging period under two temperature protocols: GHC (gradual heating and cooling) and RHC (rapid heating and cooling). (**A**) pH values. (**B**) Fructose content. (**C**) Total polyphenol content. Data are presented as mean ± SD from three independent experiments. Different letters indicate statistically significant differences within the same treatment group, as assessed by Duncan’s multiple range test (*p* < 0.05). A star symbol (*) indicates a significant difference between the two groups (GHC and RHC).

Throughout the aging process, the pH of BG consistently decreased, eventually falling below 4.5 (Figure 4A). The GHC group exhibited a more rapid and pronounced pH decline compared to the RHC group, with final pH values of 4.17 for GHC and 4.41 for RHC at the end of the aging period. The fructose content remained relatively stable until the 5th day of aging (Figure 4B), after which the trends diverged between the two groups. In the GHC group, a sharp increase in fructose content was observed on the 8th day, reaching 31.5 g/100.0 g by the 15th day. In contrast, the RHC group experienced a significant increase between the 5th and 7th days, with fructose content rising by approximately 5.3 times (*p* < 0.05). The final fructose content in the RHC group reached 19.9 g/100.0 g, which is about 63% of that in the GHC group. As shown in Figure 4C, the total polyphenolic content (TPC) also increased throughout the aging period, following a trend similar to that of fructose content. By the 15th day, the rate of increase in TPC was 3.19 times in the GHC group and 2.18 times in the RHC group compared to day 1 (Figure 4C).

### 3.4. Changes in Sulfur Compounds of Garlic Subjected to Different Temperature Conditions During Aging

**Figure 5:**
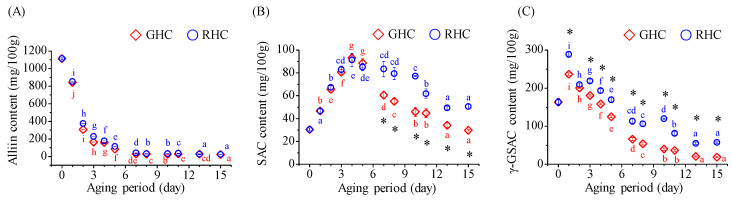
Changes in alliin, S-allylcysteine (SAC), and γ-glutamyl-S-allylcysteine (γ-GSAC) content in garlic during a 15-day aging period under two temperature protocols: GHC (gradual heating and cooling) and RHC (rapid heating and cooling). (**A**) Alliin content. (**B**) SAC content. (**C**) γ-GSAC content. Data are presented as mean ± SD from three independent experiments. Different letters indicate statistically significant differences within the same treatment group, as assessed by Duncan’s multiple range test (*p* < 0.05). A star symbol (*) indicates a significant difference between the two groups (GHC and RHC).

The changes in key sulfur compounds of garlic, including alliin, S-allylcysteine (SAC), and γ-glutamyl-S-allylcysteine (γ-GSAC), are presented in Figure 5. The alliin content showed a rapid decline within the first 5 days of aging, followed by a more gradual decrease (Figure 5A). SAC content initially peaked on the 4th day, reaching 93.3 mg/100.0 g in GHC and 91.4 mg/100.0 g in RHC (Figure 5B), representing the highest levels recorded during the aging process. After this peak, SAC levels steadily declined, with the GHC group showing a more rapid reduction. By the end of the aging period, SAC content was 29.8 mg/100.0 g in GHC and 50.5 mg/100.0 g in RHC, with the RHC group containing approximately 1.7 times higher SAC concentrations. The γ-GSAC content decreased throughout the aging period, with differing rates of reduction between GHC and RHC (Figure 5C). In GHC, γ-GSAC content dropped from 124.8 mg/100.0 g on the 5th day to 66.2 mg/100.0 g on the 7th day. In contrast, the γ-GSAC content in RHC remained above 100.0 mg/100.0 g until the 10th day of aging. By the end of the aging period, the final γ-GSAC content in RHC was 57.2 mg/100.0 g, approximately three times higher than that in GHC, demonstrating a significant difference between the two groups (*p* < 0.05).

### 3.5. PLS-DA Analysis of the Main Factors Influencing BG Quality Under Different Aging Conditions

**Figure 6:**
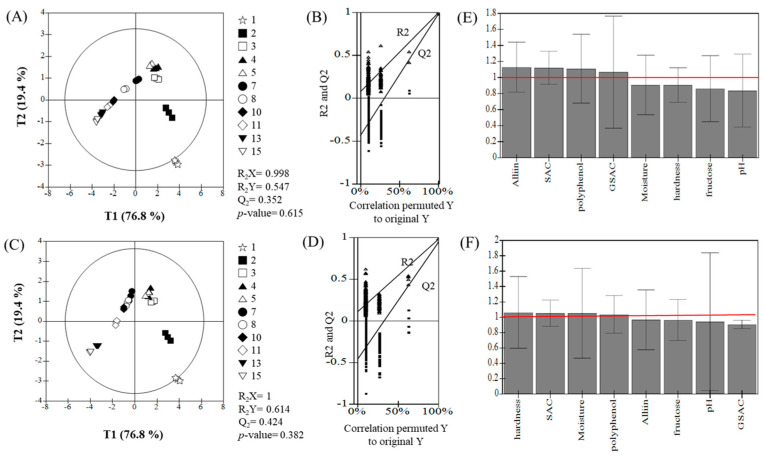
Partial Least Squares Discriminant Analysis (PLS-DA) of BG samples aged under different temperature conditions: GHC (**A**,**B**,**E**) and RHC (**C**,**D**,**F**). (**A**,**C**) PLS-DA score plots. (**B**,**D**) Permutation tests. (**E**,**F**) Variable importance in projection (VIP) scores for individual compounds. Data are cross-validated using permutation testing (*n* = 3). GHC, gradual heating and cooling. RHC, rapid heating and cooling.

Despite showing similar overall trends between the two treatment conditions, samples aged for 1 day and 10 days displayed clear distinctions from other samples along both the T1 and T2 axes (Figure 6). The PLS-DA model was validated using R_2_X, R_2_Y, Q_2_, and *p*-values, yielding the following results: GHC had R_2_X = 0.998, R_2_Y = 0.547, Q_2_ = 0.352, with a *p*-value of 0.615 (Figure 6A,B); RHC had R_2_X = 1, R_2_Y = 0.614, Q_2_ = 0.424, with a *p*-value of 0.382 (Figure 6C,D). These results indicate that the T1 factor provided the most significant differentiation between samples. The Variable Importance in Projection (VIP) values for the key factors were above 1.0, highlighting the components most influenced by the aging conditions (Figure 6E,F): for GHC, these included alliin, SAC, polyphenols, and GSAC; for RHC, they included hardness, SAC, moisture, and polyphenols. The PLS-DA analysis thus confirms that even slight differences in aging conditions can significantly impact the changes in major physicochemical components of BG.

## 4. Discussion

This study demonstrates that subtle differences in aging temperature conditions significantly impact the quality of BG. The aging process markedly alters the physical and chemical properties of garlic, with distinct variations between the two treatment groups, GHC and RHC. Although the initial and final temperatures during aging were similar, reaching peak temperatures of 85 °C and 77 °C, the rate at which these temperatures were reached—whether gradually or abruptly—was crucial in determining the levels of functional compounds in the final product.

Previous studies have shown that the physicochemical properties and bioactive compounds in garlic undergo significant changes during the BG manufacturing process [20,21,22,23]. The surface color of BG changed significantly, with reduced lightness and variations in redness and yellowness. Hardness decreased sharply, especially by day 3, resulting in a softer texture—a desirable quality for culinary and commercial appeal. Moisture content also dropped notably, with differences between treatments indicating that temperature impacts moisture retention. The pH consistently fell below 4.5, with GHC showing a faster decline, which may affect shelf life and microbial stability. These changes in color, texture, moisture, and pH are attributed to the influence of the Maillard reaction occuring during the aging process. In particular, the consistent increase in total color change (ΔE*) over the 15-day aging period for both GHC and RHC protocols highlights the significant transformations taking place in BG. By day 15, ΔE* values were similar (33.21 for GHC and 32.80 for RHC), but the rate of change differed. RHC showed a more rapid increase in the early stages, suggesting accelerated Maillard reactions due to higher peak temperatures (85 °C). GHC, with gradual temperature changes, resulted in more controlled browning. These findings indicate that RHC may be suitable for shorter production times, while GHC offers uniform transformations, highlighting their potential applications in commercial BG production.

The Maillard reaction initiates at the start of the garlic aging process, leading to an increase in organic acids—such as acetic, fumaric, malic, and citric acids—derived from carbohydrate breakdown [24,25]. The accumulation of browning compounds and organic acids lowers the pH, which in turn influences the breakdown of proteins, peptides, and polysaccharides. These interconnected reactions contribute to the distinctive black color, jelly-like texture, and sweet-sour flavor characteristic of BG [26,27]. Moisture content also plays a crucial role in developing the jelly-like texture of black garlic [6,8]. The moisture determination relates to the controlled humidity levels during BG production. High humidity (80%) in the early aging stages prevents excessive drying and hardening, while gradual reductions (to 60%, 50%, and 40%) ensure the final product achieves a moisture content of 40–50% and its characteristic jelly-like texture. Although this process is critical for texture, its direct impact on BG’s chemical composition remains unclear, requiring further research.

Our study revealed significant trends in the nutritional and functional components of BG influenced by different aging protocols. Fructose content remained stable initially but rose substantially after day 5, with a sharper increase in GHC, potentially enhancing sweetness and flavor. Total polyphenolic content, known for antioxidant benefits [28], also increased steadily, with a greater rise in GHC, suggesting higher antioxidant potential [29]. Polyphenols contribute to antioxidant and anti-inflammatory effects through the inhibition of oxidative stress and inflammatory enzymes, which may further enhance the health benefits of BG aged under GHC conditions. Sulfur compounds like alliin and γ-GSAC, beneficial for health, showed considerable reductions during aging, while SAC peaked on day 4 and then declined; RHC maintained higher SAC levels than GHC until day 15. SAC, a garlic-specific sulfur compound with potent antioxidant and anti-inflammatory properties, is often used as a quality marker for BG due to its substantial increase during aging compared to raw garlic. However, SAC is sensitive to temperature variations, peaking early and then gradually declining.

SAC is reported to be generated from γ-GSAC through transpeptidation and subsequently converted to S-alkenyl-L-cysteine sulfoxide via oxidation. Consequently, the SAC content varies depending on the extent of transpeptidation and oxidation [16,30,31]. Higher temperatures and longer aging periods may reduce transpeptidation or increase oxidation, leading to a decrease in SAC content. To better understand these changes, further studies are needed to investigate the variations in SAC content under different processing conditions, as well as the dynamics of γ-GSAC content and γ-glutamyl transpeptidase activity, which serve as precursors to SAC, during the aging process. Temperature adjustments played a key role in these compositional changes. A gradual temperature decrease from 77 °C to 59 °C favored fructose and polyphenol accumulation, while a more abrupt drop from 85 °C to 56 °C preserved higher levels of γ-GSAC and SAC. Thus, while RHC may be preferable for maximizing SAC concentration, GHC offers a balanced profile that includes enhanced polyphenols, which are known for their antioxidant and anti-inflammatory properties. These findings suggest that the choice of aging protocol should be tailored to specific product goals, such as producing BG for functional foods emphasizing SAC or for general health benefits prioritizing a balanced nutritional profile.

The antioxidant capacity of garlic is enhanced by increasing the levels of total polyphenols and organosulfur compounds such as SAC [32]. The application of steam can increase phenolic content, while prolonged heating or high pressure tends to reduce it [33]. In BG, polyphenol content can be affected by various factors, including heating conditions (temperature, pressure, time, and humidity) and aging duration. The concentration of SAC in BG is influenced by factors like heating temperature, water loss, and the activity of γ-glutamyltranspeptidase (γ-GTP). In the early stages of BG production, higher temperatures are crucial in enhancing SAC levels [34]. Our study observed that SAC concentration was significantly higher in RHC than in GHC, likely due to RHC’s initiation at a higher temperature, indicating a temperature-dependent mechanism in SAC formation. During the aging process, decreases in alliin and γ-GSAC alongside increases in SAC support this temperature effect on SAC development. Consistent with previous research, our study showed that as BG ages, there is a decrease in moisture content, pH, alliin, and γ-GSAC, while polyphenols, flavonoids, fructose, and SAC increase. These findings underscore the importance of controlled aging conditions to optimize the nutritional and functional properties of BG.

The PLS-DA and VIP data underscore the influence of temperature modulation on BG composition, particularly regarding compounds critical to flavor, antioxidant potential, and health benefits. GHC, characterized by a more gradual temperature increase, appears to favor the accumulation of polyphenols and fructose, potentially enhancing sweetness and antioxidant properties. Conversely, RHC, with its more rapid temperature adjustments, maintains higher SAC levels—a compound often highlighted in BG standardization due to its health benefits. The findings from the VIP analysis align with our recommendation for a tailored aging approach. For the standardization and commercialization of BG, it is crucial to establish a consistent aging protocol that optimizes and maintains key components like SAC, polyphenols, and flavor compounds to ensure product quality and reliability. Although this study does not establish a single standardized protocol, it provides valuable data to guide the selection of aging conditions based on intended applications. Further research is required to refine these findings into a universal protocol for BG production, balancing functional properties with commercial feasibility.

## 5. Conclusions

This study highlights the critical role of aging temperature conditions in shaping the physical and chemical characteristics of BG. GHC favored polyphenol accumulation and fructose content, enhancing BG’s antioxidant potential, whereas RHC preserved higher SAC levels, important for standardizing BG’s health benefits. These findings underscore the need for standardized aging protocols to ensure the consistent quality and bioactivity of commercial BG products, maximizing its culinary and health-related applications.

## Data Availability

The original contributions presented in the study are included in the article, further inquiries can be directed to the corresponding author.
